# Supplementary material for: Extensive androgen receptor enhancer heterogeneity in primary prostate cancers underlies transcriptional diversity and metastatic potential
Source: Nat Commun. 2022 Nov 30;13:7367. doi: 10.1038/s41467-022-35135-2 (PMC9712620; doi:10.1038/s41467-022-35135-2)
Supplement: Supplementary file 1 — Supplementary Information [file 41467_2022_35135_MOESM1_ESM.pdf]

Supplementary Information

Supplementary Figure 1

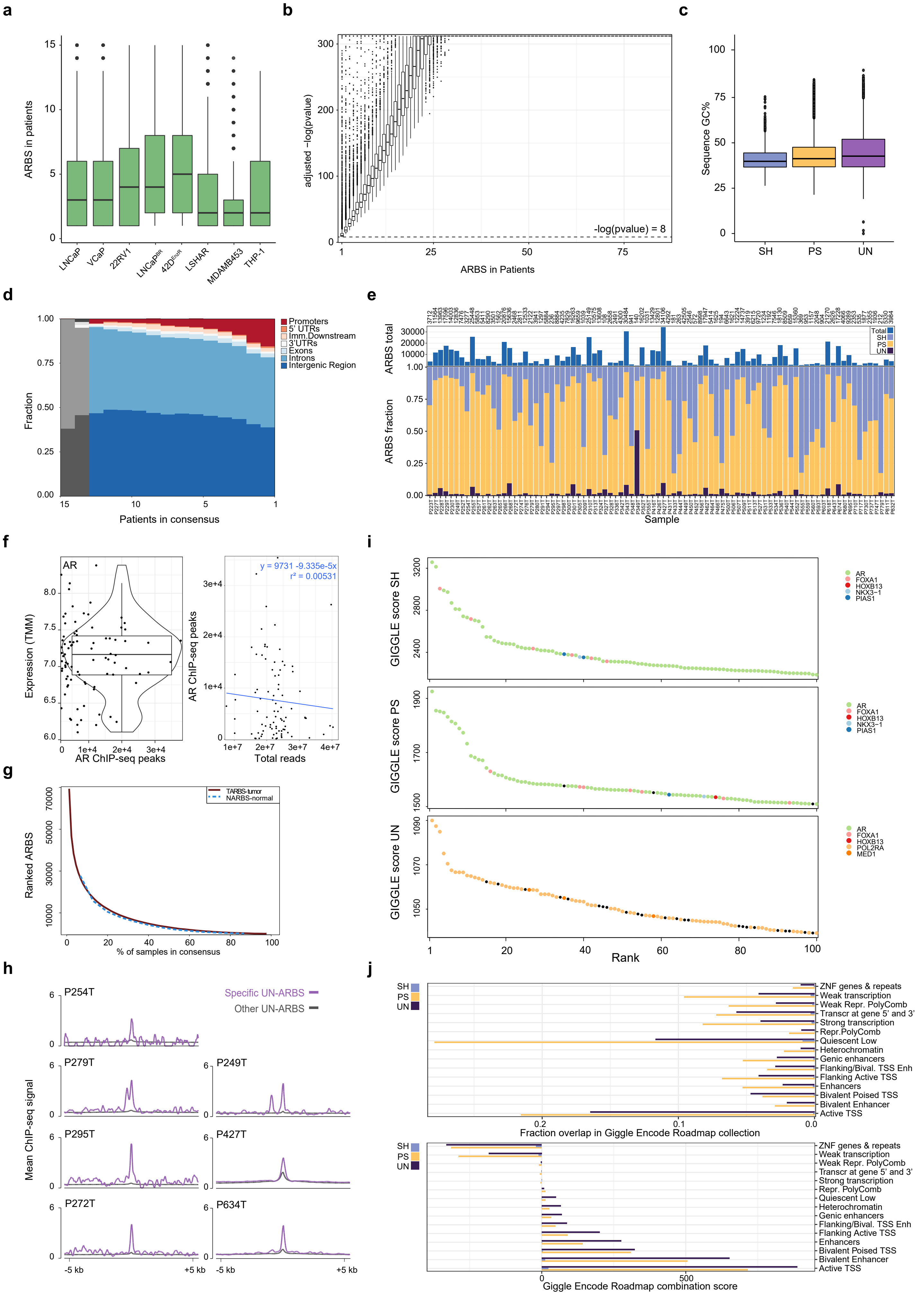

Supplementary Figure 1: Normal ranked ARBS distribution and consensus, QC, MSPC and GIGGLE analysis

(**a**) Boxplot quantification of normal tissue ranked ARBS (n=27,500) presence in cell lines. Center line, median; upper and lower quartiles; whiskers, 1.5X interquartile range; points, outliers.

(**b**) Benjamini-Hochberg adjusted –log(p-value) distributions for ranked ARBS from two-tailed Fisher's exact test by MSPC, with UN-ARBS minimum –log(p-value) of 8. Center line, median; upper and lower quartiles; whiskers, 1.5X interquartile range; points, outliers. Exact values reported in Source Data.

(**c**) Sequence nucleotide GC% for SH-ARBS, PS-ARBS and UN-ARBS. Center line, median; upper and lower quartiles; whiskers, 1.5X interquartile range; points, outliers.

(**d**) Annotated genomic consensus for normal ranked ARBS (n=27,500 from n=15 patients). Gray: instable consensus due to small number of ARBS.

(**e**) Graphical overview of ARBS total and distribution of ARBS categories (SH, PS and UN) per sample, numbers on top indicate number of peaks.

(**f**) ARBS ranking normalized for number of samples, comparing TARBS found in primary tumors (dark-red) and NARBS found in normal epithelium (blue dashes).

(**g**) Genome-wide average AR ChIP-seq intensities for tumor-specific UN-ARBS (purple) and UN-ARBS identified in all other primary tumors (87).

(**h**) Transcription Factor GIGGLE score plots for SH-ARBS, PS-ARBS and UN-ARBS for canonical PCa interactors. ChIP-seq experiments in the GIGGLE database provide no overlap of UN-ARBS for AR, FOXA1 and HOXB13, but for transcription machinery proteins POL2RA and MED1.

(**i**) GIGGLE Encode overlap and combination score of genomic locations associated with SH-ARBS, PS-ARBS and UN-ARBS in GIGGLE database.

Supplementary Figure 2

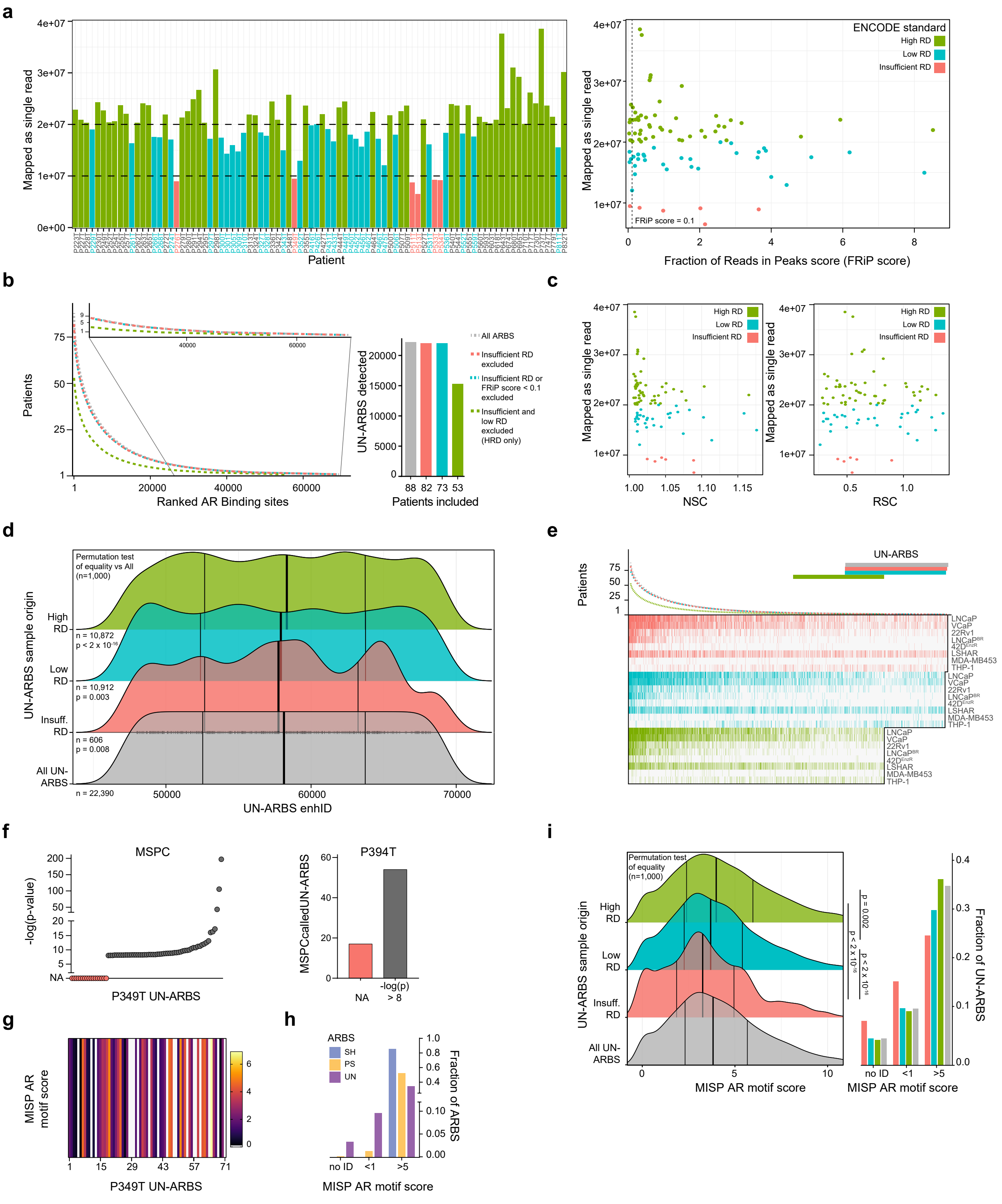

Supplementary Figure 2: AR ChIP-seq sample quality does not significantly affect ARBS ranking or UN-ARBS distributions

(**a**) Left: Overview of single mapped reads in patient samples, upper dashed line indicates 20 million reads, lower dashed line indicates 10 million reads. Right: single mapped reads plotted against FRiP score, dashed line indicates FriP score of 0.1.

(**b**) Left: Tumor ranked ARBS presence in patients for different ENCODE4 and FRiP score exclusion criteria for samples; grey = original tumor ranked ARBS, red: samples with insufficient read depth (RD), blue: samples with insufficient RD or FRiP score < 0.1, green: samples with insufficient and low RD excluded. Inset: magnification of the heterogeneous tail of ranked ARBS. Right: Barplot of UN-ARBS detected for each exclusion criterium

(**c**) Single mapped reads plotted against NSC (left) and RSC (right) for each ENCODE4 RD category.

(**d**) Density plots of UN-ARBS sample origin grouped for ENCODE4 quality classification; long lines separate quartiles, short lines indicate UN-ARBS presence from insufficient RD samples. p-values were obtained from permutation test assuming equality of distribution (n=1,000).

(**e**) Presence of ranked ARBS in various cell lines for exclusion criteria. Colored bars above indicate UN-ARBS presence.

(**f**) Left: Benjamini-Hochberg adjusted –log(pvalues) for UN-ARBS found specifically in outlier sample P394T, NA = insignificant result. Right: Barplot quantification of UN-ARBS MSPC results.

(**g**) MISP motif score for AR motifs in P394T UN-ARBS. Light grey: no match found (MISP score 0).

(**h**) Fraction of ARBS identified by MISP AR motif scoring for SH/PS/UN-ARBS grouped on score.

(**i**) Left: MISP AR motif scores for UN-ARBS grouped by ENCODE4 RD guidelines; long lines separate quartiles, p-values were obtained from permutation test assuming equality of distribution (n=1,000). Right: Fraction of UN-ARBS of total per ENCODE4 guideline category grouped by MISP AR motif scores (no ID: score = 0; <1: score <1; >5: score >5).

Supplementary Figure 3

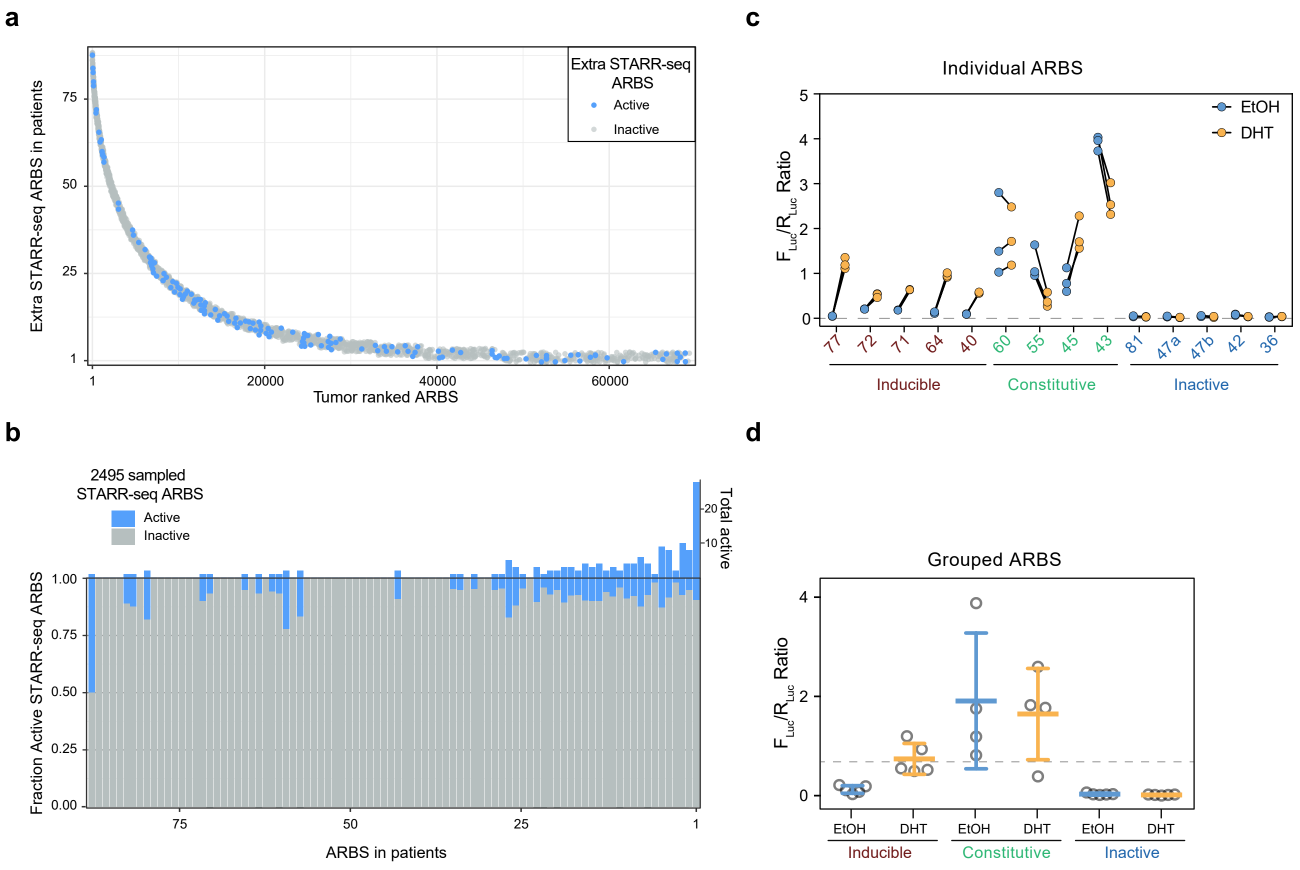

Supplementary Figure 3: Extra STARR-seq site distribution and luciferase validation

(**a**) Distribution of extra STARR-seq sites in ranked ARBS. Blue dots: extra sites with STARR-seq activity, gray dots: inactive extra sites.
(**b**) Distribution of extra STARR-seq sites with patient occurrence, top: total extra active sites per patient occurrence, extra active STARR-seq sites (blue), extra inactive STARR-seq sites (grey).
(**c**) Luciferase assay measured as ratio of Firefly luciferase over Renilla luciferase for inducible, constitutive and inactive ARBS in EtOH (vehicle, blue) vs DHT (yellow) conditions in triplicate with lines indicating triplicate pair (n = 3) after DHT stimulation. Numbers indicate occurrence of ARBS in patients out of 88.
(**d**) Grouped representation of Firefly luciferase over Renilla luciferase ratio for EtOH and DHT conditions of minimum of four experiments of triplicates (n = 3). Center line, median; upper and lower quartiles; whiskers, 1.5X interquartile range, points: mean of luciferase assays per ARBS. Error bars denote SD

Supplementary Figure 4

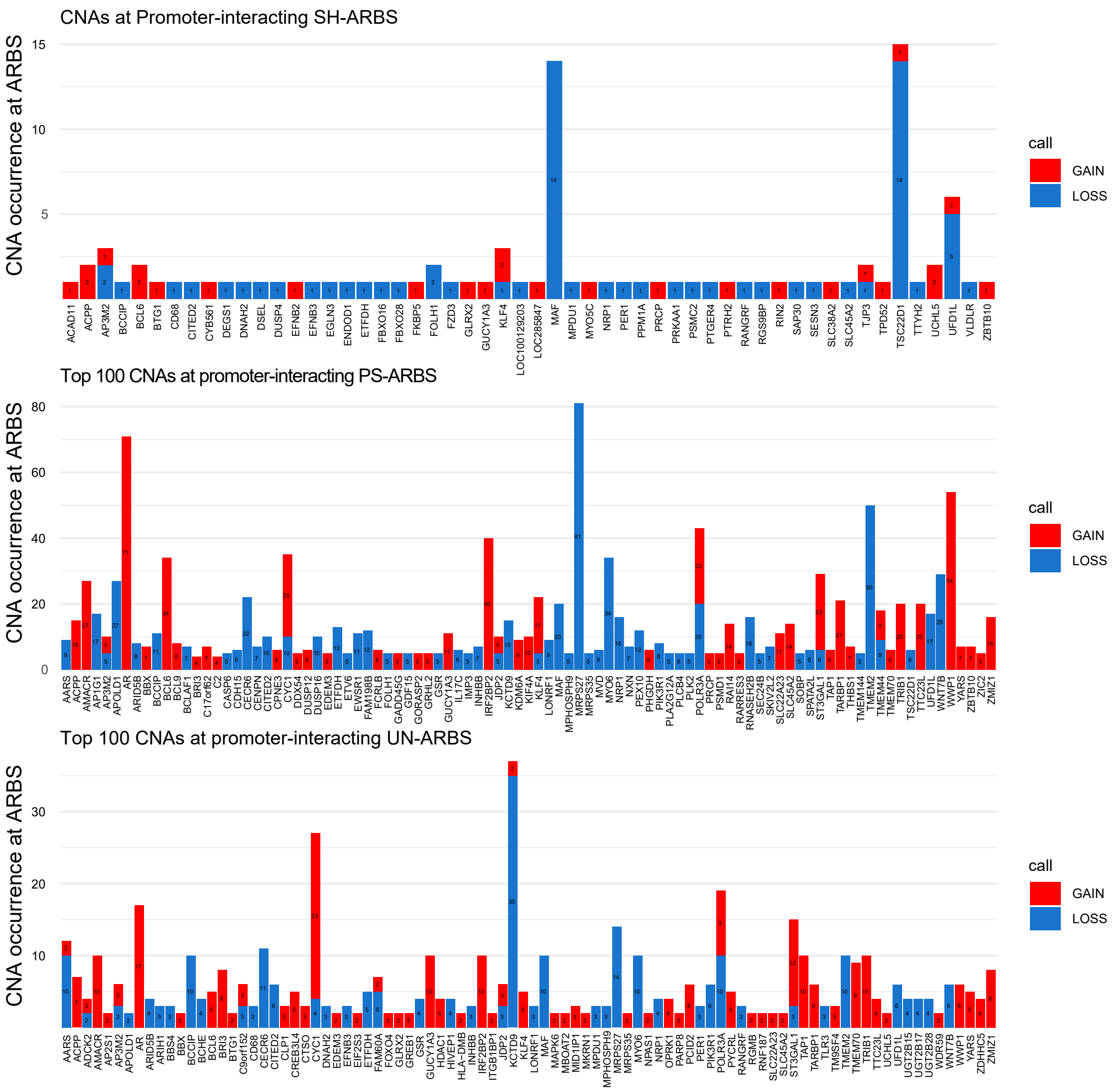

Supplementary Figure 4: Copy number gains and losses at promoter interacting ARBS

CNA occurrence at promoter-interacting SH-ARBS, PS-ARBS and UN-ARBS showing that most promoter-interacting ARBS are predominantly affected by either copy number gains or losses. Top 100 most-occuring CNAs are plotted for PS-ARBS and UN-ARBS, numbers on bars indicate CNA occurrence.

Supplementary Figure 5

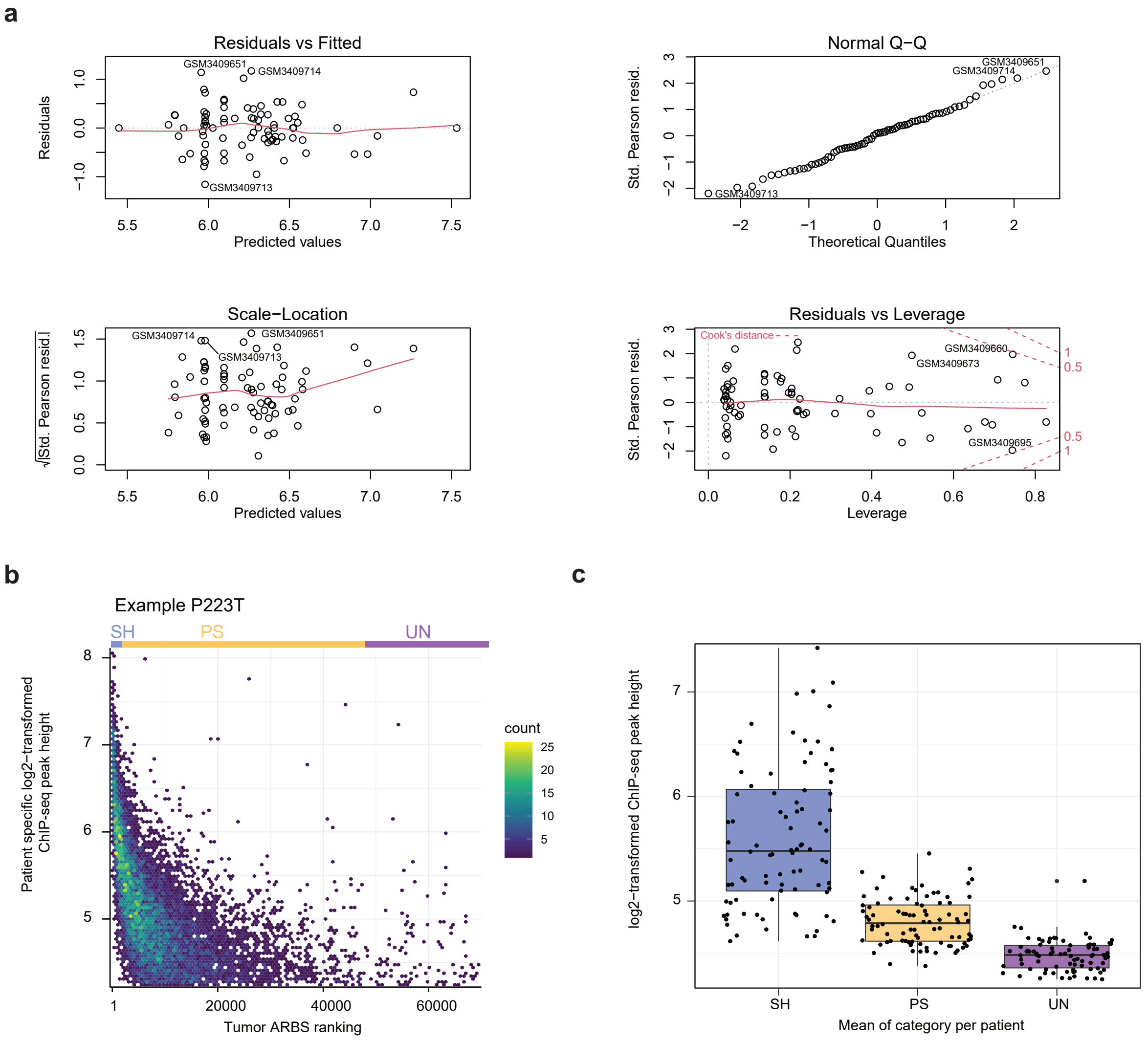

Supplementary Figure 5: GLM assumption tests and inverse ChIP-seq peak height correlation per category

(**a**) CITED2 GLM assumption plots clockwise from top left: residuals versus fitted values plot, linearity testing in normal Q-Q plot, residuals versus leverage plot with Cook's distance plotted in red, scale-location plot. Outlying patients are labelled in the plots.

(**b**) Example plot of sample P223T detailing AR log2-transformed ChIP-seq peak height and ARBS ranking, points are averaged per patient after SH/PS/UN-ARBS binning.

(**c**) Boxplot of average AR log2-transformed ChIP-seq peak height per patient (dots) per ARBS category. Center line, median; upper and lower quartiles; whiskers, 1.5X interquartile range; points, outliers.

Supplementary Figure 6

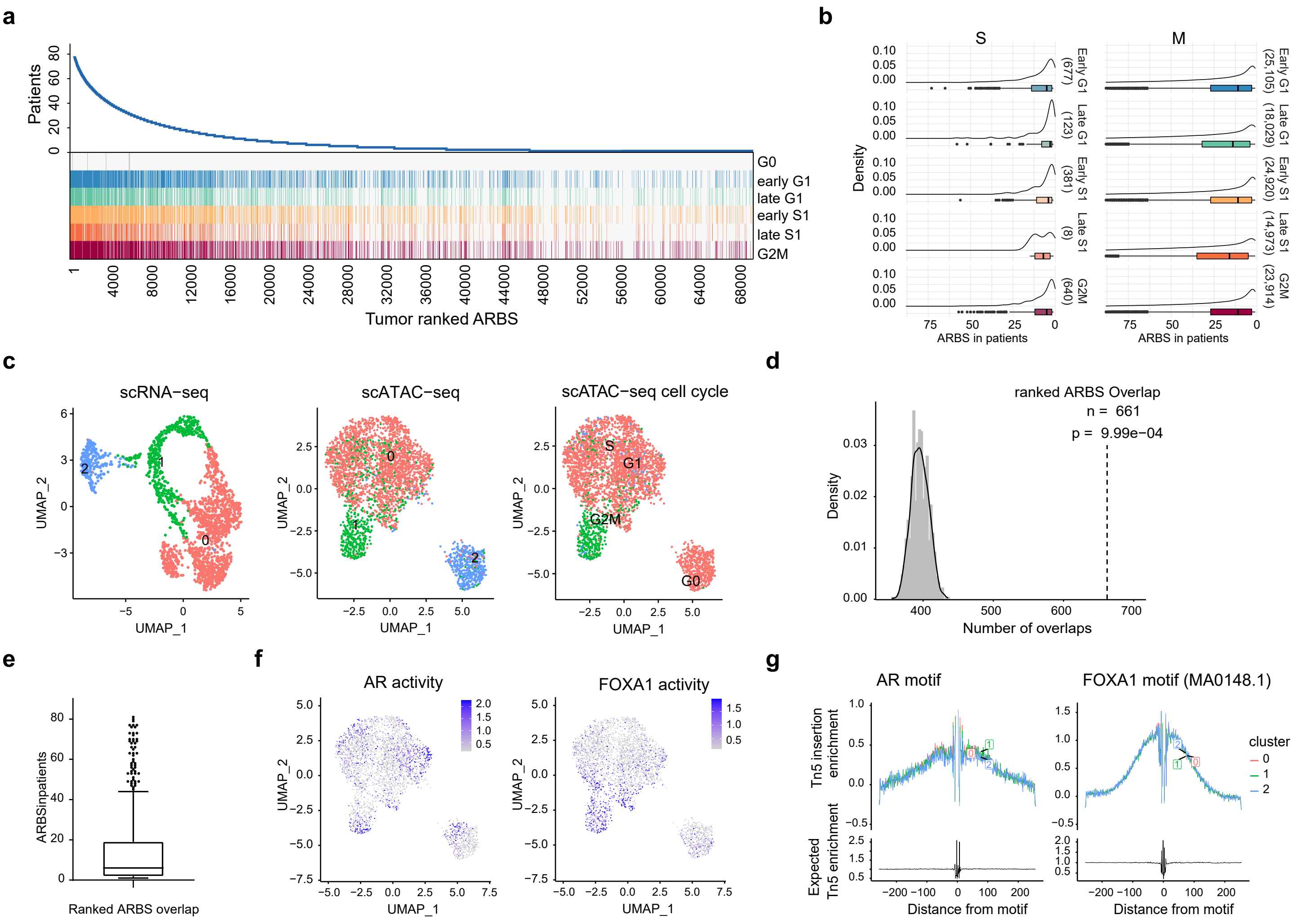

Supplementary Figure 6: Heterogeneity of cell cycle specific ARBS and LNCaP chromatin accessibility

(**a**) Presence of ARBS from cell cycle phase arrested bulk LNCaP in ranked ARBS.

(**b**) Distribution of cell-cycle specific ARBS in patients for sites found specifically in a specific cell cycle (S) or sites found in multiple cell cycles (M). Number of sites (n = ARBS) detailed in parentheses. Center line, median; upper and lower quartiles; whiskers, 1.5X interquartile range; points, outliers.

(**c**) UMAP plots of LNCaP-DMSO scRNA-seq with three distinct clusters (left), LNCaP-DMSO scATAC-seq with the three scRNA-seq cluster labels overlaid (middle) and scATAC-seq with cell cycle phases labels as inferred by gene accessibility and scRNAseq.

(**d**) Density plot of permutation tests of ARBS overlap enrichment at differentially accessible sites versus all background peaks (grey), n = 661 ARBS, p = 9.99e-4, 1,000 permutations.

(**e**) Differentially accessible ARBS overlap with ranked tumor ARBS for prevalence in patients. Center line, mean; upper and lower quartiles; whiskers, 1.5X interquartile range; points, outliers.

(**f**) AR (left) and FOXA1 (right) activity scores overlaid over scATAC-seq UMAP plots.

(**g**) De novo motif search in differentially accessible peaks showing observed (top) and expected (bottom) Tn5 insertion enrichment around AR and FOXA1 (MA0148.1) motifs among clusters.

Supplementary Figure 7

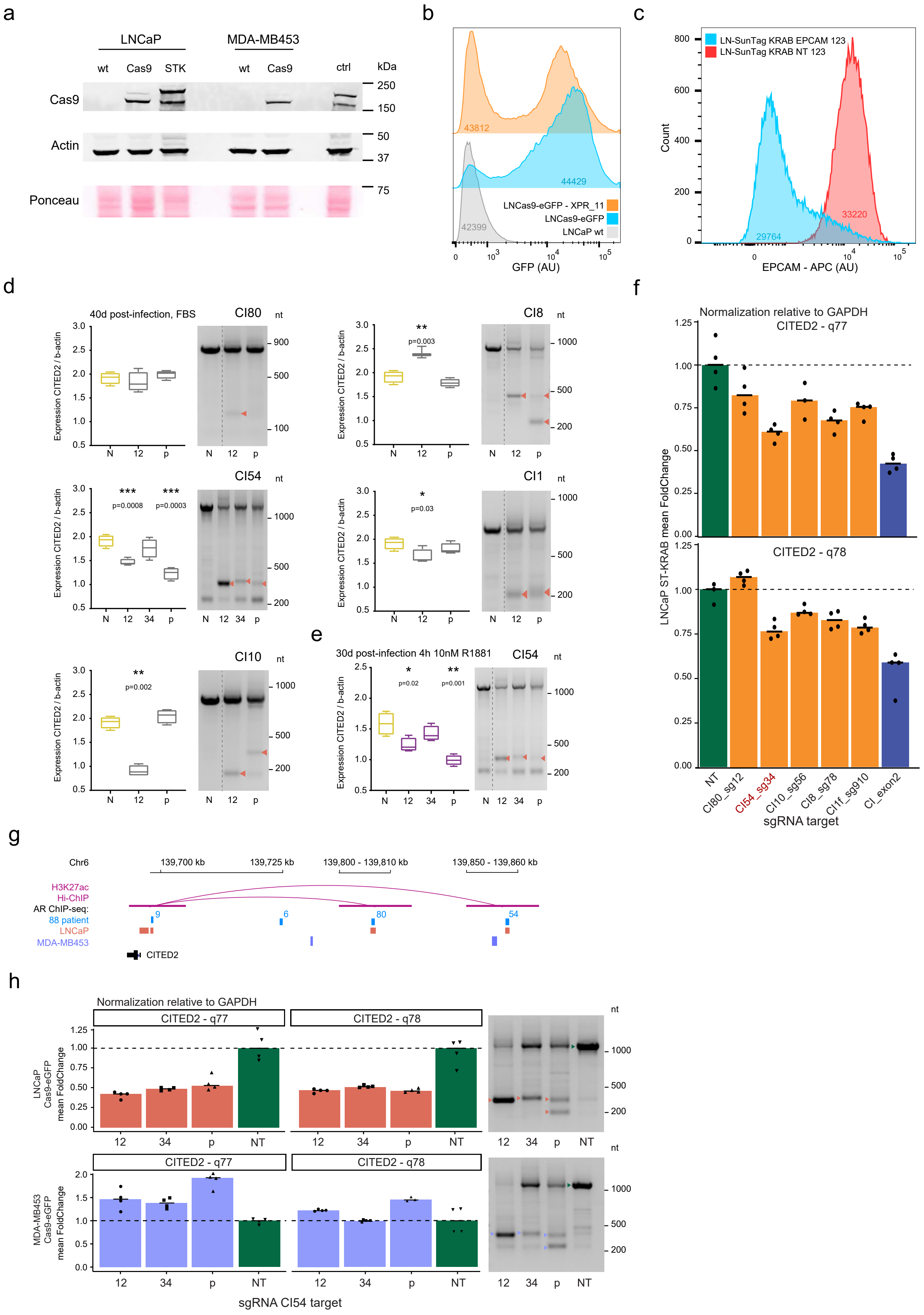

Supplementary Figure 7: Validation experiments for Cas9-mediated enhancer cuts and repression using Suntag-KRAB

(**a**) Western blot for Cas9 and actin for Cas9-eGFP infected LNCaP and MDA-MB-453 cells and LNCaP Suntag-KRAB. Control: Cas9+ cell line, loading control actin. Representative experiment of 3 independent blots.

(**b**) Flow cytometry histograms of GFP for LNCaP wild-type (gray), LNCaP:Cas9-eGFP with NT sgRNA (blue) and LNCaP:Cas9-eGFP with GFP sgRNA (orange). Numbers indicate cell counts.

(**c**) Flow cytometry histograms of EPCAM-APC for LNCaP:Suntag-KRAB with NT sgRNA pool (red) and LNCaP:Suntag-KRAB with EPCAM sgRNA pool (light blue). Numbers indicate cell counts.

(**d**) Replicate qPCR of CITED2 over beta-actin and gDNA PCRs of Cas9-mediated ARBS cutting for H3K27ac Hi-ChIP interacting CITED2 enhancers 40 days post-infection in FBS. Center line, median; upper and lower quartiles; whiskers, 1.5X interquartile range, nt = nucleotide weight.

(**e**) Independent validation of CITED2 expression over beta-actin for Cas9-mediated cut out of CITED2-interacting ARBS found in 54 patients. Cells harvested 30 days post-infection after 3 days of hormonal deprivation and R1881 stimulation. Center line, median; upper and lower quartiles; whiskers, 1.5X interquartile range. Two-tailed Student t-test of means, $p < 0.05$ *, $p < 0.01$ **, $p < 0.001$ ***, nt = nucleotide weight.

(**f**) LNCaP:Suntag-KRAB qPCR of NT normalized CITED2 over GAPDH for H3K27ac HiChIP interacting CITED2 enhancers and CITED2 exon 2 targeting sgRNAs at 7 days post-infection using two different qPCR primer pairs (q77 and q78). Center line indicates median,

(**g**) Genomic snapshot of CITED2 locus at Chr6 with H3K27ac HiChIP interactions and AR ChIP-seq of 88 primary patients (blue), LNCaP cells (red) and AR^+ breast cancer cell line MDA-MB-453 (purple). Blue numbers indicate number of patients in which ARBS was identified, nt = nucleotide weight.

(**h**) LNCaP:Cas9-eGFP qPCR of NT sgRNA normalized CITED2 over GAPDH and gDNA verification PCRs of Cas9-mediated ARBS cutting for H3K27ac HiChIP interacting CITED2 enhancers 40 days post-infection in FBS. Center line indicates median, nt = nucleotide weight. Arrows indicate expected bands as result of successful CRISPR pair KO.

Supplementary Figure 8

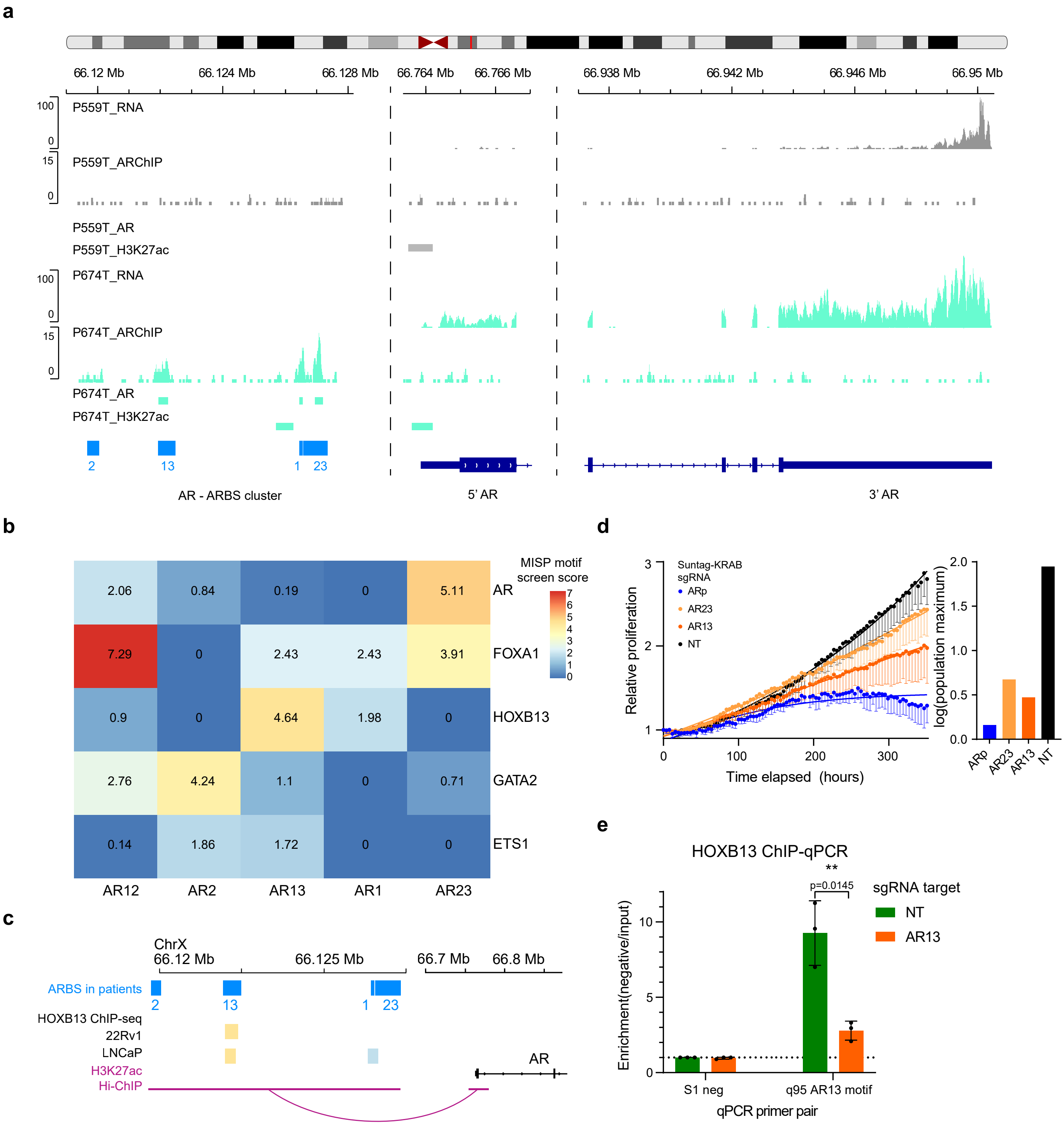

Supplementary Figure 8: AR somatic enhancer binding in primary patients and TF motif enrichment

(**a**) IGV genomic snapshot of upstream AR locus and AR coding sequence loci with AR ChIP-seq, H3K27ac ChIP-seq and matched RNA-seq for two selected patient tissues from primary cohort.
(**b**) Motif enrichment scores of AR, FOXA1, HOXB13, GATA2 and ETS1 for ARBS found in 12, 2, 13, 1 and 23 patients.
(**c**) Genomic snapshot of AR enhancer locus and AR promoter, showing ranked ARBS (occurrence in blue numbers), HOXB13 ChIP-seq for 22Rv1 and LNCaP cells color-coded for MISP-motif score as in (**b**) and H3K27ac HIChIP interaction data.
(**d**) Left: LNCaP:Suntag-KRAB proliferation for sgRNAs targeted at AR promoter (ARp), enhancers AR23, AR13 and Non-targeting (NT) sgRNA. Data are presented as mean values +/- SD.
(**e**) HOXB13 ChIP-qPCR of HOXB13 enrichment at AR13 over negative control (S1). Mean of minimum two biological replicates (n=2), Data are presented as mean values +/- SD. Center line, median; upper and lower quartiles; whiskers, 1.5X interquartile range.

Supplementary Figure 9

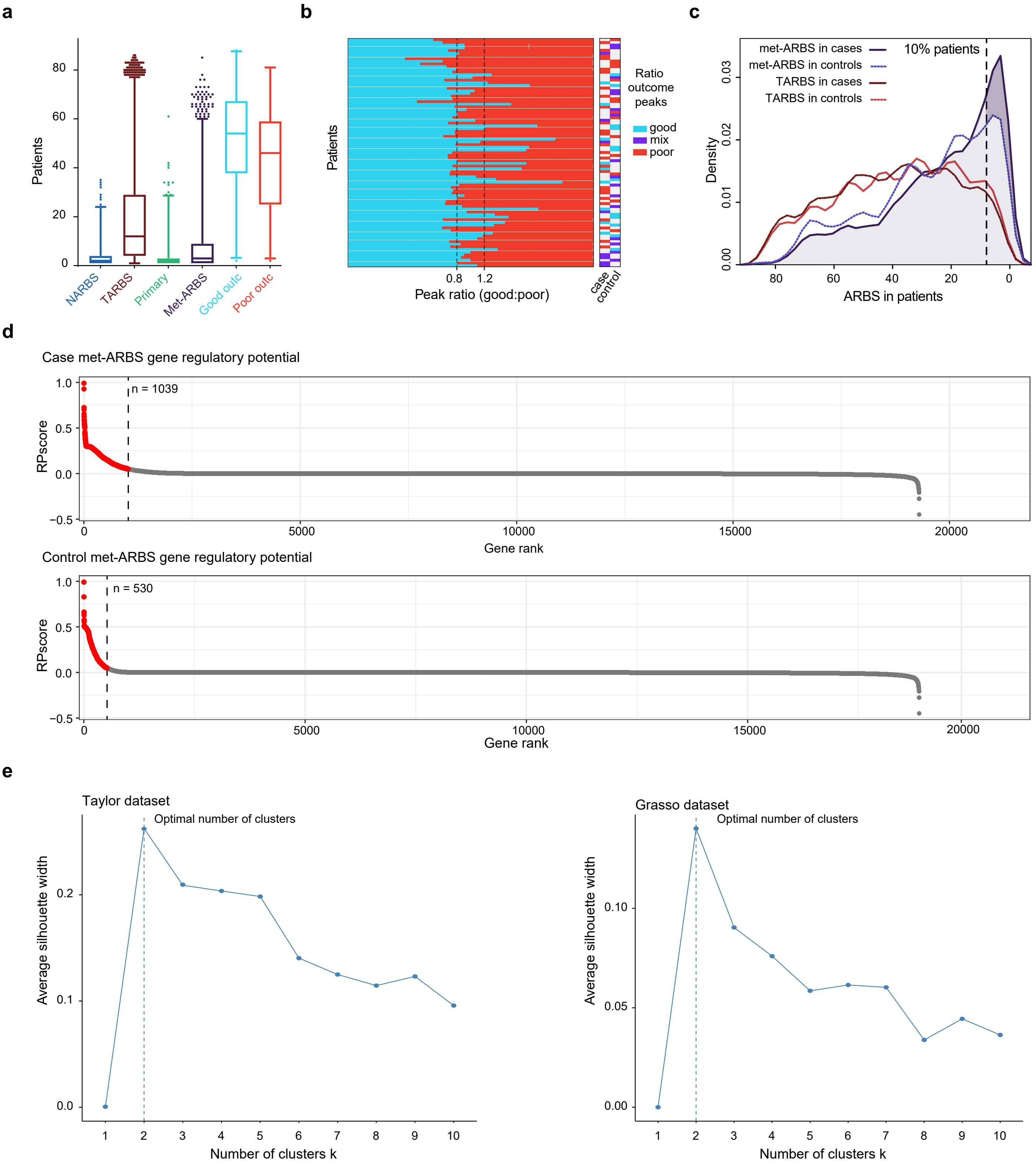

Supplementary Figure 9: Quantification of PCa state-specific ARBS, GO RP scores and Silhouette plots

(**a**) Distribution of PCa state-specific ARBS among primary tumor ranked ARBS. Center line, median; upper and lower quartiles; whiskers, 1.5X interquartile range; points, outliers.

(**b**) Left: Overview of good outcome (blue) and bad (red) outcome ARBS distributions of all patients. Dashed lines indicate cut-off for ratio good outcome/poor outcome peaks. Right: Ratio outcome peaks designation based on cut-offs between case and control patients defined by BCR, with patients with equal ratios of good and poor outcome peaks denoted by purple (mix).

(**c**) Density plots of cumulative occurrence of TARBS and met-ARBS for cases and controls in ranked ARBS. Dotted line indicates ARBS found in < 10% patients, with dark purple fill-in highlighting the difference between cases and controls.

(**d**) GO Regulatory Potential score ranking of genes for met-ARBS identified in 8 or less case (n=1,024) and control (n=526) primary patients, red: RP score cut-off >0.05.

(**e**) Silhouette plots for determining optimal k means clustering for Taylor and Grasso PCa tissue expression datasets.

Supplementary Table 1: Overview of used databases with accession numbers

Study accession overview table indicating what public data was used.

First author	Senior author	Year	Ref.	DOI	Publication	Accession	Source	Type	Comment	ENCODE QC guideline (out of 6)
Stelloo	Zwart	2018	3	10.1038/s41467-018-07270-2	Nat Commun	GSE70079	Tissue	Primary	Ranked ARBS, matched AR ChIP-seq and RNAseq and outcome on BCR	
Pomerantz	Freedman	2015	4	10.1038/ng.3419	Nat Gen	GSE56288	Tissue	Normal	N-ARBS, combined with Pomerantz et al. 2020 for normal ranking	
Pomerantz	Freedman	2015	4	10.1038/ng.3419	Nat Gen	GSE56288	Tissue	Epithelial	T-ARBS	
Pomerantz, Qiu, Zhu	Freedman	2020	5	10.1038/s41588-020-0664-8	Nat Gen	GSE130408	Tissue	Normal	Normal ARBS ranking 8 + 7 samples from N-ARBS	
Pomerantz, Qiu, Zhu	Freedman	2020	5	10.1038/s41588-020-0664-8	Nat Gen	GSE130408	PDX	mCRPC	Met-ARBS	6
Takayama	Inoue	2015	16	10.1038/ncomms9219	Nat Commun	GSE66037	Cells	LNCaP-BicR	AR ChIP-seq (combined with own LNCaP ChIP-seq)	6
Toropainen	Palvimo	2016	17	10.1038/srep33510	Sci Rep	GSE84432	Cells	VCaP	AR ChIP-seq (combined with Kron et al.)	6
Kron	Lupien	2017	15	10.1038/ng.3930	Nat Gen	GSE56086	Cells	VCaP	AR ChIP-seq (combined with Toropainen et al.)	6
Kron	Lupien	2017	15	10.1038/ng.3930	Nat Gen	GSE56086	Cells	22Rv1	AR ChIP-seq	6
Pomerantz	Freedman	2015	4	10.1038/ng.3419	Nat Gen	GSE56288	Cells	LSHAR	AR ChIP-seq (combined with own LSHAR ChIP-seq)	6
Cioni	Bergman	2020	18	10.1038/s41467-020-18313-y	Nat Commun	GSE131381	Cells	THP-1	AR ChIP-seq	6
Davies	Zoubeidi	2021	54	10.1038/s41556-021-00743-5	Nat Cell Biol	GSE138460	Cells	42D-ENZR	AR ChIP-seq	6
Malinen	Palvimo	2015	19	10.1016/j.mce.2015.07.024	Mol Cell Endocrinol	GSE70161	Cells	MDA-MB453	AR ChIP-seq	6

Huang	Lack	2021	12	10.1186/s13059-021-02339-6	Genome Biol	GSE151064	Cells	LNCaP	STARR-seq 286 inducible, 463 constitutive,<br>2467 inact, 1237 pred. ind, 1681 cons	
Zhang	Wang	2018	76	10.1038/s41467-018-04451-x	Nat Commun	Available upon request			PCa-specific rSNP	
Schumacher,<br>Olama	GAME-ON &ELLIPSE consortia	2018	77	10.1038/s41588-018-0142-8	Nat Gen	phs001391.v1.p1			PCa-specific rSNP	
DeRycke	Thibodeau	2019	78	10.1371/journal.pone.0214588	PLOS one	phs000985.v1.p1			PCa-specific rSNP	
Pomerantz,<br>Qiu, Zhu	Freedman	2020	5	10.1038/s41588-020-0664-8	Nat Gen	GSE130408			PCa-specific rSNP	
Fraser	Boutros	2017	10	10.1038/nature20788	Nature	GSE84043	Tissue	Primary	pSNV affected ARBS, 200 WGS and 277 WES	
Quigley	Feng	2018	11	10.1016/j.cell.2018.06.039	Cell	Author site	Tissue	mCRPC	mSNV affected ARBS, 101 metastatic samples	
Jiang	Li	2019	9	10.1093/nar/gky1025	Nucleic Acids Res	-		Various cell lines and tissue	Super-enhancer mapping database	
Khan	Zhang	2016	76	10.1093/nar/gkv1002	Nucleic Acids Res			LNCaP	Super-enhancer mapping database	
Stelloo	Zwart	2015	8	10.15252/emmm.201505424	EMBO Mol Med	GSE65478	Tissue	normal, primary, ADT	138 good, 197 poor outcome sites	
Pomerantz,<br>Giambartolomei	Freedman	2020	5	10.1016/j.ajhg.2021.11.007	Nat Gen, AJHG	GSE130408	Cells	LNCaP	H3K27ac Hi-ChIP chromatin interaction	
Taavitsainen	Urbanucci	2021	14	10.1038/s41467-021-25624-1	Nat Commun	GSE168733	Cells	LNCaP	scATAC-seq	
Takeda	Freedman	2018	13	10.1016/j.cell.2018.05.037	Cell	GSE114385	Tissue	mCRPC	149 tumors from 60 men,<br>AR enhancer sgRNA tiling screen	
Taylor	Gerald	2010	48	10.1016/j.ccr.2010.05.026	Cancer Cell	GSE21032	Tissue	Primary and metastatic	218 PCa tumor mRNA expression and outcome	
Grasso	Tomlins	2012	49	10.1038/nature11125	Nature	GSE35988	Tissue	Primary and metastatic	50 mCRPC and 11 treatment-naïve primary expression	
McNair	Knudsen	2016	41	10.1038/onc.2016.334	Oncogene	GSE82201	Cells	LNCaP	AR ChIP-seq of cell-cycle arrested LNCaP + DHT	6

Supplementary Table 2: Primary prostate cancer tissue tumor cell percentage

Pathologist assessment of primary prostate cancer tissues

Sample ID	% tumor cells	group_key
P223T	80-90%	2
P227T	70-80%	3
P228T	80-90%	2
P229T	80-90%	3
P230T	80-90%	3
P249T	70-80%	2
P252T	80-90%	2
P254T	80-90%	2
P255T	70-80%	3
P257T	80-90%	2
P261T	70-80%	3
P262T	70-80%	3
P263T	70-80%	3
P265T	70-80%	3
P266T	70-80%	3
P268T	70-80%	3
P272T	80-90%	2
P274T	80-90%	2
P276T	80-90%	2
P279T	80-90%	2
P280T	70-80%	3
P291T	70-80%	3
P294T	80-90%	2
P295T	70-80%	3
P297T	80-90%	2
P298T	80-90%	2
P300T	80-90%	2
P301T	80-90%	2
P305T	70-80%	3
P309T	80-90%	2
P310T	80-90%	2
P313T	80-90%	2
P324T	70-80%	3
P327T	70-80%	3
P328T	70-80%	3
P336T	80-90%	2
P342T	70-80%	3
P343T	80-90%	2

P348T	70-80%	3
P349T	70-80%	3
P352T	80-90%	2
P355T	80-90%	2
P416T	70-80%	3
P426T	70-80%	3
P427T	80-90%	2
P431T	80-90%	2
P433T	80-90%	2
P444T	70-80%	3
P449T	80-90%	2
P450T	70-80%	3
P452T	70-80%	3
P456T	80-90%	2
P462T	80-90%	2
P464T	80-90%	2
P466T	80-90%	2
P475T	70-80%	3
P500T	80-90%	2
P506T	80-90%	2
P507T	80-90%	2
P509T	70-80%	3
P511T	80-90%	2
P513T	70-80%	3
P527T	80-90%	2
P531T	80-90%	2
P533T	80-90%	2
P534T	80-90%	2
P536T	70-80%	3
P540T	70-80%	3
P544T	70-80%	3
P552T	80-90%	2
P555T	80-90%	2
P559T	80-90%	2
P560T	80-90%	2
P593	80-90%	2
P603	70-80%	3
P618	70-80%	3
P643	70-80%	3
P674	90-100%	1
P680	80-90%	2

P695	80-90%	2
P710	80-90%	2
P717	70-80%	3
P730	70-80%	3
P737	80-90%	2
P747	80-90%	2
P779	70-80%	3
P811	70-80%	3
P832	80-90%	2

Supplementary Table 3: AR ChIP-seq peaks identified per tissue sample

Called peaks and accession numbers for used AR-ChIP-seq.

Sample	ARpeaks_total	SH	PS	UN	Full_acc
P223T	3712	1108	2585	19	GSM3409642_P223T
P227T	11564	1190	10154	220	GSM3409643_P227T
P228T	13963	1183	11987	793	GSM3409644_P228T
P229T	17596	1190	15845	561	GSM3409645_P229T
P230T	14003	1201	12679	123	GSM3409646_P230T
P249T	12863	1200	11504	159	GSM3409647_P249T
P252T	7476	1122	6294	60	GSM3409648_P252T
P254T	3277	1132	2144	1	GSM3409649_P254T
P255T	25448	1182	22592	1674	GSM3409650_P255T
P257T	5853	1112	4642	99	GSM3409651_P257T
P261T	5411	1164	4237	10	GSM3409652_P261T
P262T	8290	1178	7027	85	GSM3409653_P262T
P263T	3501	1129	2364	8	GSM3409654_P263T
P265T	1562	762	786	14	GSM3409655_P265T
P266T	20876	1193	19323	360	GSM3409656_P266T
P268T	15636	1176	13006	1454	GSM3409657_P268T
P272T	2468	1037	1425	6	GSM3409658_P272T
P274T	2811	995	1798	18	GSM3409659_P274T
P276T	12533	1193	11141	199	GSM3409660_P276T
P279T	2122	964	1151	7	GSM3409661_P279T
P280T	3941	1118	2805	18	GSM3409662_P280T
P291T	1297	800	494	3	GSM3409663_P291T
P294T	5884	1198	4673	13	GSM3409664_P294T
P295T	236	176	57	3	GSM3409665_P295T
P297T	8884	1190	7600	94	GSM3409666_P297T
P298T	4235	1139	3091	5	GSM3409667_P298T

P300T	7829	1166	6558	105	GSM3409668_P300T
P301T	16263	1179	13672	1412	GSM3409669_P301T
P305T	9659	1193	8332	134	GSM3409670_P305T
P309T	1039	648	389	2	GSM3409671_P309T
P310T	25149	1197	22703	1249	GSM3409672_P310T
P313T	10575	1186	9304	85	GSM3409673_P313T
P324T	13608	1194	12149	265	GSM3409674_P324T
P327T	108	74	29	5	GSM3409675_P327T
P328T	2658	1004	1605	49	GSM3409676_P328T
P336T	2491	1017	1468	6	GSM3409677_P336T
P342T	4300	1021	3181	98	GSM3409678_P342T
P343T	30484	1189	26844	2451	GSM3409679_P343T
P348T	941	578	353	10	GSM3409680_P348T
P349T	140	15	54	71	GSM3409681_P349T
P352T	16202	1190	14834	178	GSM3409682_P352T
P355T	1031	657	371	3	GSM3409683_P355T
P416T	13429	1199	12152	78	GSM3409684_P416T
P426T	12801	1196	11408	197	GSM3409685_P426T
P427T	34106	1181	29609	3316	GSM3409686_P427T
P431T	4292	1115	3162	15	GSM3409687_P431T
P433T	181	150	28	3	GSM3409688_P433T
P444T	263	178	77	8	GSM3409689_P444T
P449T	4205	1121	3065	19	GSM3409690_P449T
P450T	2546	1019	1512	15	GSM3409691_P450T
P452T	572	334	232	6	GSM3409692_P452T
P456T	8998	1192	7743	63	GSM3409693_P456T
P462T	17947	1189	15695	1063	GSM3409694_P462T
P464T	5414	1137	4220	57	GSM3409695_P464T
P466T	1625	872	740	13	GSM3409696_P466T
P475T	194	145	39	10	GSM3409697_P475T
P500T	6643	1165	5367	111	GSM3409698_P500T
P506T	1621	869	747	5	GSM3409699_P506T
P507T	12224	1191	10770	263	GSM3409700_P507T
P509T	14737	1197	13292	248	GSM3409701_P509T
P511T	3919	1124	2755	40	GSM3409702_P511T
P513T	6215	1136	4919	160	GSM3409703_P513T
P527T	9720	1189	8475	56	GSM3409704_P527T
P531T	1234	590	638	6	GSM3409705_P531T
P533T	1192	656	527	9	GSM3409706_P533T
P534T	7546	1137	6281	128	GSM3409707_P534T
P536T	18130	1193	16318	619	GSM3409708_P536T

P540T	8509	1198	7298	13	GSM3409709_P540T
P544T	659	464	192	3	GSM3409710_P544T
P552T	15360	1194	13483	683	GSM3409711_P552T
P555T	369	304	62	3	GSM3409712_P555T
P559T	953	653	297	3	GSM3409713_P559T
P560T	1157	674	478	5	GSM3409714_P560T
P593T	2048	989	1056	3	GSM3409715_P593T
P603T	904	568	331	5	GSM3409716_P603T
P618T	21270	1181	18951	1138	GSM3409717_P618T
P643T	2626	1124	1500	2	GSM3409718_P643T
P674T	17228	1194	14527	1507	GSM3409719_P674T
P680T	4066	1021	3015	30	GSM3409720_P680T
P695T	9289	1179	7907	203	GSM3409721_P695T
P710T	2353	709	1617	27	GSM3409722_P710T
P717T	535	387	142	6	GSM3409723_P717T
P730T	1877	942	932	3	GSM3409724_P730T
P737T	2355	986	1368	1	GSM3409725_P737T
P747T	1936	805	1124	7	GSM3409726_P747T
P779T	212	175	32	5	GSM3409727_P779T
P811T	5330	1116	4136	78	GSM3409728_P811T
P832T	3984	973	2947	64	GSM3409729_P832T

Supplementary Table 4: Sequencing read quality control metrics

Sequencing read quality control metrics including negative strand correlation (NSC), relative strand correlation (RSC), peak number and fraction of reads in peaks score (FRiP).

Sample_ID	Factor (Antibody)	Patient_ID	Case_Control	nr reads	fraction mapped	mapped as single read	NSC	RSC	Peak number	FRiP score
P223T_AR	AR (Santa Cruz; sc-816)	P223T	Cases	24620474	0.926469653	22810123	1.014255	0.5828583	3741	0.59
P227T_AR	AR (Santa Cruz; sc-816)	P227T	Cases	22532537	0.925737523	20859216	1.031401	0.8259269	12385	2.15
P228T_AR	AR (Santa Cruz; sc-816)	P228T	Control	22393694	0.906389942	20297420	1.04292	0.8170973	15065	2.75
P229T_AR	AR (Santa Cruz; sc-816)	P229T	Cases	20102780	0.944982933	18996785	1.083032	1.125217	18039	3.8
P230T_AR	AR (Santa Cruz; sc-816)	P230T	Cases	25470608	0.954118253	24301973	1.050791	1.019307	14473	2.46
P249T_AR	AR (Santa Cruz; sc-816)	P249T	Control	24092278	0.940599722	22661191	1.072363	1.146209	13533	2.53
P252T_AR	AR (Santa Cruz; sc-816)	P252T	Control	21827155	0.933125091	20367467	1.035776	0.7780387	7676	1.43
P254T_AR	AR (Santa Cruz; sc-816)	P254T	Exclude	22065358	0.931446931	20552711	1.012942	0.4856578	3299	0.49
P255T_AR	AR (Santa Cruz; sc-816)	P255T	Cases	39760895	0.595352796	23671761	1.10351	1.162961	26294	5.93
P257T_AR	AR (Santa Cruz; sc-816)	P257T	Control	24398018	0.90297966	22030915	1.01918	0.6574779	6228	0.95
P261T_AR	AR (Santa Cruz; sc-816)	P261T	Cases	17134997	0.952968652	16329116	1.021821	0.590407	5462	0.98
P262T_AR	AR (Santa Cruz; sc-816)	P262T	Control	22083425	0.917859571	20269484	1.029478	0.708004	8525	1.51
P263T_AR	AR (Santa Cruz; sc-816)	P263T	Control	25254852	0.952162618	24046727	1.011729	0.5730436	3520	0.5
P265T_AR	AR (Santa Cruz; sc-816)	P265T	Cases	24537475	0.965765141	23697439	1.010187	0.4820882	1631	0.21
P266T_AR	AR (Santa Cruz; sc-816)	P266T	Control	18391065	0.952325599	17514283	1.102531	1.250378	21530	5.12
P268T_AR	AR (Santa Cruz; sc-816)	P268T	Cases	18530102	0.942160167	17458325	1.055521	0.9800846	16440	3.62
P272T_AR	AR (Santa Cruz; sc-816)	P272T	Control	22882964	0.957330265	21906555	1.013207	0.4651862	2506	0.35
P274T_AR	AR (Santa Cruz; sc-816)	P274T	Cases	18531555	0.919200143	17034209	1.023941	0.6022613	2905	0.47
P276T_AR	AR (Santa Cruz; sc-816)	P276T	Cases	10230637	0.873984875	8941423	1.088797	0.8957601	12649	3.65
P279T_AR	AR (Santa Cruz; sc-816)	P279T	Control	22890081	0.932096833	21335773	1.013752	0.5239322	2135	0.33
P280T_AR	AR (Santa Cruz; sc-816)	P280T	Control	24308849	0.925808581	22505342	1.018043	0.6037517	4013	0.61
P291T_AR	AR (Santa Cruz; sc-816)	P291T	Exclude	26473609	0.93913006	24862163	1.009063	0.4102503	1308	0.2
P294T_AR	AR (Santa Cruz; sc-816)	P294T	Cases	27577000	0.966994815	26666817	1.015899	0.7373516	5975	0.88
P295T_AR	AR (Santa Cruz; sc-816)	P295T	Cases	21782551	0.932503039	20312296	1.007714	0.29512	243	0.06
P297T_AR	AR (Santa Cruz; sc-816)	P297T	Control	20423475	0.842618213	17209193	1.032097	0.6877458	9292	1.74
P298T_AR	AR (Santa Cruz; sc-816)	P298T	Cases	31717776	0.965477025	30622785	1.011609	0.6345308	4277	0.62
P300T_AR	AR (Santa Cruz; sc-816)	P300T	Control	18024314	0.964227376	17379538	1.024965	0.7435726	7930	1.49
P301T_AR	AR (Santa Cruz; sc-816)	P301T	Cases	15337785	0.93041003	14270430	1.074537	1.040754	17971	4

P305T_AR	AR (Santa Cruz; sc-816)	P305T	Control	17057286	0.933874475	15929365	1.033817	0.7666989	9721	1.77
P309T_AR	AR (Santa Cruz; sc-816)	P309T	Cases	15343434	0.959416125	14720739	1.010899	0.3537316	1053	0.19
P310T_AR	AR (Santa Cruz; sc-816)	P310T	Cases	19842529	0.922866989	18312016	1.158604	1.350412	25931	6.19
P313T_AR	AR (Santa Cruz; sc-816)	P313T	Cases	23622319	0.925976954	21873724	1.040194	1.097381	10685	2.05
P324T_AR	AR (Santa Cruz; sc-816)	P324T	Control	27881638	0.780162055	21752197	1.034603	0.7534276	14256	2.44
P327T_AR	AR (Santa Cruz; sc-816)	P327T	Cases	22873723	0.804624765	18404765	1.010837	0.2930086	112	0.04
P328T_AR	AR (Santa Cruz; sc-816)	P328T	Control	21117434	0.839515871	17728422	1.019486	0.4815153	2835	0.45
P336T_AR	AR (Santa Cruz; sc-816)	P336T	Control	25665987	0.950061106	24384257	1.011008	0.5393414	2532	0.36
P342T_AR	AR (Santa Cruz; sc-816)	P342T	Control	22714184	0.917901431	20849383	1.015971	0.5750244	4428	0.7
P343T_AR	AR (Santa Cruz; sc-816)	P343T	Cases	16790768	0.891348091	14966420	1.175974	1.284918	32286	8.28
P348T_AR	AR (Santa Cruz; sc-816)	P348T	Cases	37451664	0.686169538	25698192	1.008018	0.3310427	956	0.13
P349T_AR	AR (Santa Cruz; sc-816)	P349T	Control	16625464	0.570367239	9482621	1.044588	0.5297713	159	0.05
P352T_AR	AR (Santa Cruz; sc-816)	P352T	Cases	16801279	0.770909167	12952261	1.113918	1.121093	16598	4.43
P355T_AR	AR (Santa Cruz; sc-816)	P355T	Cases	24516545	0.964314221	23641654	1.009308	0.4370284	1037	0.14
P416T_AR	AR (Santa Cruz; sc-816)	P416T	Control	21707438	0.913058879	19820170	1.064851	1.078288	13569	2.88
P426T_AR	AR (Santa Cruz; sc-816)	P426T	Cases	27969311	0.714637268	19987913	1.040421	0.7639348	13213	2.58
P427T_AR	AR (Santa Cruz; sc-816)	P427T	Control	23331954	0.941921924	21976880	1.165201	1.287467	35444	8.52
P431T_AR	AR (Santa Cruz; sc-816)	P431T	Cases	20023371	0.952344338	19069145	1.015911	0.5586526	4320	0.72
P433T_AR	AR (Santa Cruz; sc-816)	P433T	Control	17673974	0.945158174	16704702	1.008366	0.28976	187	0.04
P444T_AR	AR (Santa Cruz; sc-816)	P444T	Control	33080874	0.702958332	23254477	1.008699	0.3178689	280	0.04
P449T_AR	AR (Santa Cruz; sc-816)	P449T	Cases	25850491	0.943894489	24400137	1.015309	0.659915	4265	0.75
P450T_AR	AR (Santa Cruz; sc-816)	P450T	Cases	19632834	0.914069665	17945779	1.015074	0.4567794	2604	0.43
P452T_AR	AR (Santa Cruz; sc-816)	P452T	Cases	18301541	0.940094881	17205186	1.008353	0.3298432	586	0.08
P456T_AR	AR (Santa Cruz; sc-816)	P456T	Control	16474488	0.949295905	15639165	1.044092	0.9712267	9139	1.96
P462T_AR	AR (Santa Cruz; sc-816)	P462T	Cases	19416843	0.956555965	18573298	1.067002	1.111203	18152	3.85
P464T_AR	AR (Santa Cruz; sc-816)	P464T	Cases	23412496	0.95579177	22377472	1.015796	0.6188879	5444	0.9
P466T_AR	AR (Santa Cruz; sc-816)	P466T	Control	23740042	0.725033427	17212325	1.012576	0.3166301	1637	0.28
P475T_AR	AR (Santa Cruz; sc-816)	P475T	Control	16396532	0.735520719	12059990	1.014086	0.2615917	197	0.11
P500T_AR	AR (Santa Cruz; sc-816)	P500T	Cases	22743775	0.957414238	21775215	1.02012	0.7443276	6988	1.18
P506T_AR	AR (Santa Cruz; sc-816)	P506T	Cases	18892336	0.95161276	17978189	1.011238	0.3894433	1635	0.28

P507T_AR	AR (Santa Cruz; sc-816)	P507T	Control	23640431	0.952227055	22511059	1.050234	1.089082	12527	2.69
P509T_AR	AR (Santa Cruz; sc-816)	P509T	Control	24838040	0.950150777	23599884	1.045322	1.027597	15205	2.87
P511T_AR	AR (Santa Cruz; sc-816)	P511T	Control	10169031	0.858821553	8733384	1.035416	0.4732332	3981	0.98
P513T_AR	AR (Santa Cruz; sc-816)	P513T	Control	7600645	0.854784693	6496916	1.089194	0.5451723	6471	2.15
P527T_AR	AR (Santa Cruz; sc-816)	P527T	Cases	23525604	0.889926822	20936067	1.034908	0.8335207	9778	1.73
P531T_AR	AR (Santa Cruz; sc-816)	P531T	Cases	17387106	0.925676015	16094828	1.011333	0.3573592	1244	0.19
P533T_AR	AR (Santa Cruz; sc-816)	P533T	Control	10137898	0.908096136	9206187	1.024668	0.4177768	1197	0.32
P534T_AR	AR (Santa Cruz; sc-816)	P534T	Control	10344994	0.881695533	9121136	1.050203	0.58669	7717	2.02
P536T_AR	AR (Santa Cruz; sc-816)	P536T	Cases	19451396	0.944123908	18364529	1.056687	1.03814	18517	3.65
P540T_AR	AR (Santa Cruz; sc-816)	P540T	Control	24787062	0.965102439	23922055	1.022654	0.8363826	8581	1.34
P544T_AR	AR (Santa Cruz; sc-816)	P544T	Control	24497185	0.963530014	23603774	1.008138	0.4303424	670	0.09
P552T_AR	AR (Santa Cruz; sc-816)	P552T	Cases	19634450	0.926693185	18195112	1.053509	0.9875975	15685	3.03
P555T_AR	AR (Santa Cruz; sc-816)	P555T	Control	24564693	0.964565199	23694249	1.008108	0.4398692	372	0.06
P559T_AR	AR (Santa Cruz; sc-816)	P559T	Cases	18546627	0.948469067	17590903	1.010282	0.412166	979	0.15
P560T_AR	AR (Santa Cruz; sc-816)	P560T	Control	22990170	0.932291888	21433550	1.008363	0.3770089	1176	0.19
P593T_AR	AR (Santa Cruz; sc-816)	P593T	Control	21569433	0.946475181	20414934	1.010544	0.4512739	2056	0.31
P603T_AR	AR (Santa Cruz; sc-816)	P603T	Control	22122389	0.909496981	20120247	1.008241	0.3296038	910	0.12
P618T_AR	AR (Santa Cruz; sc-816)	P618T	Cases	22496530	0.92879893	20894754	1.07833	1.149379	22334	4.83
P643T_AR	AR (Santa Cruz; sc-816)	P643T	Cases	39410557	0.954011992	37598145	1.008729	0.5514827	2645	0.38
P674T_AR	AR (Santa Cruz; sc-816)	P674T	Control	23990337	0.963961073	23125752	1.061178	1.214612	17570	3.17
P680T_AR	AR (Santa Cruz; sc-816)	P680T	Control	32039846	0.967549688	31000144	1.01152	0.651814	4115	0.63
P695T_AR	AR (Santa Cruz; sc-816)	P695T	Control	30185677	0.967926709	29217524	1.019941	0.8212429	9377	1.5
P710T_AR	AR (Santa Cruz; sc-816)	P710T	Cases	21053335	0.953726761	20079130	1.010814	0.4853518	2371	0.36
P717T_AR	AR (Santa Cruz; sc-816)	P717T	Cases	27656933	0.945459245	26148504	1.005989	0.2997338	540	0.08
P730T_AR	AR (Santa Cruz; sc-816)	P730T	Cases	25477648	0.944103239	24053531	1.009375	0.4354283	1894	0.28
P737T_AR	AR (Santa Cruz; sc-816)	P737T	Cases	40315672	0.95573637	38531155	1.007202	0.4819284	2367	0.33
P747T_AR	AR (Santa Cruz; sc-816)	P747T	Control	25064139	0.942388885	23620167	1.009182	0.4016096	1954	0.29
P779T_AR	AR (Santa Cruz; sc-816)	P779T	Control	23151291	0.925237431	21420442	1.006052	0.2822717	216	0.03
P811T_AR	AR (Santa Cruz; sc-816)	P811T	Cases	22317858	0.69529876	15517580	1.023737	0.4960241	5482	1.08
P832T_AR	AR (Santa Cruz; sc-816)	P832T	Control	31799628	0.948560467	30163871	1.010932	0.5420255	4147	0.6

Supplementary Table 5: Total tumor ranked ARBS identified in cell lines

Identified tumor ranked ARBS per AR-ChIP-seq cell line dataset

ARBS overlap	Tumor	Normal
EnzR_42D	2117	1075
LNBR	5980	3486
LNCAP	28105	10449
VCAP	24270	9848
22RV1	9403	3794
LSHAR	21943	13960
MDAMB453	1046	3866
THP-1	7462	422

Supplementary Table 6: Luciferase validation of STARR-seq

Overview of assayed STARR-seq ARBS in luciferase validation experiment, including locus, enhID and patient prevalence.

chr	start	end	EtOH_Rep 1	EtOH_Rep 2	EtOH_Rep 3	DHT_Rep 1	DHT_Rep 2	DHT_Rep 3	enhID	patients	Classification	Region
chr15	99346784	99347415	0.129579982	0.110749186	0.141772152	0.917870722	0.943234837	1.013771997	881	64	Inducible	2022-ARBS
chr13	77629275	77629836	0.099483823	0.093511087	0.093104036	0.56368434	0.558885925	0.584075505	3815	40	Inducible	434-ARBS
chr12	47186625	47187264	0.052362805	0.045936825	0.050319197	1.356236625	1.109414576	1.187467919	215	77	Inducible	1413-ARBS
chr17	34148294	34148884	0.207704799	0.209264042	0.204826647	0.545322685	0.537096807	0.466615575	1995	72	Inducible	789-ARBS
chr11	34692562	34693146	0.235960238	0.230558235	0.225129781	0.549161476	0.532444522	0.537889354	5124	34	Inducible	559-ARBS
chr1	10695822	10696501	3.73084753	4.034394432	3.960286649	2.316693768	2.533351699	3.022447372	3240	43	Constitutive	254-ARBS
chr7	86973621	86974175	0.779571601	0.600813317	1.127591241	1.561161872	1.70711317	2.280861761	2896	45	Constitutive	72-ARBS
chr2	237906442	237907527	0.957694637	1.038722503	1.635756057	0.270188877	0.365211028	0.582504441	1623	55	Constitutive	1791-ARBS
chr9	117670263	117670916	1.030121717	1.494792795	2.802194692	1.186594042	1.714205819	2.482880322	755	60	Constitutive	2220-ARBS
chr2	153158557	153159194	0.093897755	0.068651525	0.081650033	0.03733557	0.034932625	0.043552088	3387	42	Inactive	416-ARBS
chr2	23968519	23969094	0.023605827	0.034862776	0.029906555	0.041842416	0.035905024	0.042799543	4639	36	Inactive	469-ARBS
chr2	215259605	215260264	0.041315127	0.036496948	0.054085844	0.037596024	0.026994587	0.032186209	89	81	Inactive	661-ARBS
chr2	79344574	79345178	0.035029072	0.031500267	0.047548748	0.016123835	0.016494502	0.022189893	2560	47	Inactive	2102-ARBS (47a)
chr11	74088679	74089252	0.044581746	0.03998185	0.060440622	0.026107944	0.021728414	0.042867508	2542	47	Inactive	2702-ARBS (47b)

Supplementary Table 7: Copy Number Alterations at the upstream AR enhancer locus

Identified copy number gains at upstream AR enhancer locus with patient ID.

chr	start	end	patid	call
chrX	66073001	66701000	DTB-156-BL	GAIN
chrX	65107001	66188000	DTB-069-BL	GAIN
chrX	66047001	66294000	DTB-126-BL	GAIN
chrX	65758001	66263000	DTB-176-BL	GAIN
chrX	65729001	66496000	DTB-265-PRO	GAIN
chrX	64895001	66749000	DTB-175-BL	GAIN
chrX	62515001	66310000	DTB-091-BL	GAIN
chrX	66097001	66351000	DTB-092-BL	GAIN
chrX	57742001	66158000	DTB-071-BL	GAIN
chrX	65525001	66580000	DTB-005-BL	GAIN
chrX	65616501	66478000	DTB-137-PRO	GAIN
chrX	65474001	66192000	DTB-024-PRO	GAIN
chrX	56351001	66621000	DTB-205-BL	GAIN
chrX	63801001	66320000	DTB-037-BL	GAIN
chrX	65393001	66449000	DTB-102-PRO	GAIN
chrX	58499001	66755000	DTB-214-BL	GAIN
chrX	65700001	66687000	DTB-089-BL	GAIN
chrX	65919001	65993000	DTB-186-BL	GAIN
chrX	64866001	66026000	DTB-085-BL	GAIN
chrX	64309001	66024000	DTB-151-BL	GAIN
chrX	65621001	66097000	DTB-092-BL	GAIN
chrX	65800001	66047000	DTB-126-BL	GAIN
chrX	58499001	66035000	DTB-216-PRO	GAIN

Supplementary Table 8: DepMap IDs used for accession of CRISPR KO databases

Accession codes for genome-wide KO screens deposited in https://depmap.org/portal/.

cell line	DB	dep_id
22Rv1	Sanger	ACH-000956
LNCaP	GeCKO_19Q1 & Sanger	ACH-000977
VCAP	Avana_20Q1	ACH-000115

Supplementary Table 9: Outcome ARBS identified in primary prostate cancer patients

Ratio of good versus poor outcome sites per patient and class stratification definition.

Sample	GSM_number	good	poor	case_ctrl	gp_ratio	class
P223T	GSM3409642	96	86	case	1.116	mix
P227T	GSM3409643	116	145	case	0.800	mix
P228T	GSM3409644	131	131	control	1.000	mix
P229T	GSM3409645	136	159	case	0.855	mix
P230T	GSM3409646	131	146	case	0.897	mix
P249T	GSM3409647	131	131	control	1.000	mix
P252T	GSM3409648	128	82	control	1.561	good
P255T	GSM3409650	129	180	case	0.717	poor
P257T	GSM3409651	88	104	control	0.846	mix
P261T	GSM3409652	91	130	case	0.700	poor
P262T	GSM3409653	116	104	control	1.115	mix
P263T	GSM3409654	95	78	control	1.218	good
P265T	GSM3409655	52	36	case	1.444	good
P266T	GSM3409656	133	185	control	0.719	poor
P268T	GSM3409657	106	170	case	0.624	poor
P272T	GSM3409658	87	59	control	1.475	good
P274T	GSM3409659	84	47	case	1.787	good
P276T	GSM3409660	101	161	case	0.627	poor
P279T	GSM3409661	78	41	control	1.902	good
P280T	GSM3409662	94	63	control	1.492	good
P294T	GSM3409664	111	119	case	0.933	mix
P295T	GSM3409665	14	4	case	3.500	good
P297T	GSM3409666	117	135	control	0.867	mix
P298T	GSM3409667	74	113	case	0.655	poor
P300T	GSM3409668	95	143	control	0.664	poor
P301T	GSM3409669	107	134	case	0.799	poor
P305T	GSM3409670	122	135	control	0.904	mix
P309T	GSM3409671	33	45	case	0.733	poor
P310T	GSM3409672	134	178	case	0.753	poor
P313T	GSM3409673	113	156	case	0.724	poor
P324T	GSM3409674	126	152	control	0.829	mix
P327T	GSM3409675	7	1	case	7.000	good
P328T	GSM3409676	81	53	control	1.528	good
P336T	GSM3409677	69	63	control	1.095	mix
P342T	GSM3409678	67	95	control	0.705	poor
P343T	GSM3409679	134	188	case	0.713	poor

P348T	GSM3409680	51	21	case	2.429	good
P352T	GSM3409682	130	176	case	0.739	poor
P355T	GSM3409683	59	22	case	2.682	good
P416T	GSM3409684	126	157	control	0.803	mix
P426T	GSM3409685	111	161	case	0.689	poor
P427T	GSM3409686	127	186	control	0.683	poor
P431T	GSM3409687	78	106	case	0.736	poor
P433T	GSM3409688	8	3	control	2.667	good
P444T	GSM3409689	17	6	control	2.833	good
P449T	GSM3409690	71	91	case	0.780	poor
P450T	GSM3409691	75	64	case	1.172	mix
P452T	GSM3409692	50	9	case	5.556	good
P456T	GSM3409693	102	161	control	0.634	poor
P462T	GSM3409694	129	167	case	0.772	poor
P464T	GSM3409695	87	113	case	0.770	poor
P466T	GSM3409696	57	45	control	1.267	good
P475T	GSM3409697	17	5	control	3.400	good
P500T	GSM3409698	94	137	case	0.686	poor
P506T	GSM3409699	55	51	case	1.078	mix
P507T	GSM3409700	130	176	control	0.739	poor
P509T	GSM3409701	130	160	control	0.813	mix
P511T	GSM3409702	76	96	control	0.792	poor
P513T	GSM3409703	97	104	control	0.933	mix
P527T	GSM3409704	116	151	case	0.768	poor
P531T	GSM3409705	60	30	case	2.000	good
P533T	GSM3409706	25	64	control	0.391	poor
P534T	GSM3409707	97	131	control	0.740	poor
P536T	GSM3409708	124	172	case	0.721	poor
P540T	GSM3409709	133	134	control	0.993	mix
P544T	GSM3409710	31	29	control	1.069	mix
P552T	GSM3409711	121	179	case	0.676	poor
P555T	GSM3409712	23	8	control	2.875	good
P559T	GSM3409713	45	34	case	1.324	good
P560T	GSM3409714	29	45	control	0.644	poor
P593T	GSM3409715	60	58	control	1.034	mix
P603T	GSM3409716	41	29	control	1.414	good
P618T	GSM3409717	131	176	case	0.744	poor
P643T	GSM3409718	66	84	case	0.786	poor
P674T	GSM3409719	119	177	control	0.672	poor
P680T	GSM3409720	58	139	control	0.417	poor
P695T	GSM3409721	106	169	control	0.627	poor

P710T	GSM3409722	26	86	case	0.302	poor
P717T	GSM3409723	22	28	case	0.786	poor
P730T	GSM3409724	48	58	case	0.828	mix
P737T	GSM3409725	53	77	case	0.688	poor
P747T	GSM3409726	60	66	control	0.909	mix
P779T	GSM3409727	10	11	control	0.909	mix
P811T	GSM3409728	75	120	case	0.625	poor
P832T	GSM3409729	52	98	control	0.531	poor
