# Supplementary material for: Extensive androgen receptor enhancer heterogeneity in primary prostate cancers underlies transcriptional diversity and metastatic potential
Source: Nat Commun. 2022 Nov 30;13:7367. doi: 10.1038/s41467-022-35135-2 (PMC9712620; doi:10.1038/s41467-022-35135-2)
Supplement: Supplementary file 3 — Description of Additional Supplementary Files [file 41467_2022_35135_MOESM3_ESM.pdf]

Description of Additional Supplementary Files

File Name: Supplementary Data 1
Description: Overview of oligonucleotides used in study
