# Supplementary material for: Extensive androgen receptor enhancer heterogeneity in primary prostate cancers underlies transcriptional diversity and metastatic potential
Source: Nat Commun. 2022 Nov 30;13:7367. doi: 10.1038/s41467-022-35135-2 (PMC9712620; doi:10.1038/s41467-022-35135-2)
Supplement: Supplementary file 5 — Reporting Summary [file 41467_2022_35135_MOESM5_ESM.pdf]

nature portfolio

Corresponding author(s): Wilbert Zwart, Andre Bergman

Last updated by author(s): 31/10/2022

Reporting Summary

Nature Portfolio wishes to improve the reproducibility of the work that we publish. This form provides structure for consistency and transparency in reporting. For further information on Nature Portfolio policies, see our Editorial Policies and the Editorial Policy Checklist.

Statistics

For all statistical analyses, confirm that the following items are present in the figure legend, table legend, main text, or Methods section.

n/a	Confirmed	
☐	☒	The exact sample size (n) for each experimental group/condition, given as a discrete number and unit of measurement
☐	☒	A statement on whether measurements were taken from distinct samples or whether the same sample was measured repeatedly
☐	☒	The statistical test(s) used AND whether they are one- or two-sided<br>*Only common tests should be described solely by name; describe more complex techniques in the Methods section.*
☐	☒	A description of all covariates tested
☐	☒	A description of any assumptions or corrections, such as tests of normality and adjustment for multiple comparisons
☐	☒	A full description of the statistical parameters including central tendency (e.g. means) or other basic estimates (e.g. regression coefficient) AND variation (e.g. standard deviation) or associated estimates of uncertainty (e.g. confidence intervals)
☐	☒	For null hypothesis testing, the test statistic (e.g. F, t, r) with confidence intervals, effect sizes, degrees of freedom and P value noted<br>*Give P values as exact values whenever suitable.*
☐	☒	For Bayesian analysis, information on the choice of priors and Markov chain Monte Carlo settings
☐	☒	For hierarchical and complex designs, identification of the appropriate level for tests and full reporting of outcomes
☐	☒	Estimates of effect sizes (e.g. Cohen's d, Pearson's r), indicating how they were calculated

Our web collection on statistics for biologists contains articles on many of the points above.

Software and code

Policy information about availability of computer code

Data collection	NCBI GEO was used to obtain data from publicly accessible databases, which are comprehensively listed in Supplementary Table 1.
Data analysis	R(v4.1.1), MACS(v1.4), BEDtoolsv(2.29), Easeq(v1.03), MSPC(v5.4.0), NMF(v0.23.0), DiffBind(v3.4), ChIPpeakAnno(v3.28.0), DESeq(v1.32.0), GRanges(v1.47.0), Seurat(v4.0.5), Signac(v1.4.0), rtracklayer (v1.54.0),chromVar(v1.16.0), Cicero(v1.12.0), monocle3(v1.1.0), GSEA(v4.1.0), MSigDB(v7.3), factoextra(v1.0.7), qvalue(v2.28.0), sm(v2.2) code availabilty statement provided in manuscript.<br>Code is available through https://github.com/jknp/arbshet

For manuscripts utilizing custom algorithms or software that are central to the research but not yet described in published literature, software must be made available to editors and reviewers. We strongly encourage code deposition in a community repository (e.g. GitHub). See the Nature Portfolio guidelines for submitting code & software for further information.

Data

Policy information about availability of data

All manuscripts must include a data availability statement. This statement should provide the following information, where applicable:

- Accession codes, unique identifiers, or web links for publicly available datasets
- A description of any restrictions on data availability
- For clinical datasets or third party data, please ensure that the statement adheres to our policy

Data availability statement provided in manuscript.

Field-specific reporting

Please select the one below that is the best fit for your research. If you are not sure, read the appropriate sections before making your selection.

- [x] Life sciences
- [ ] Behavioural & social sciences
- [ ] Ecological, evolutionary & environmental sciences

For a reference copy of the document with all sections, see nature.com/documents/nr-reporting-summary-flat.pdf

Life sciences study design

All studies must disclose on these points even when the disclosure is negative.

Sample size	Ranked ARBS were taken from earlier published work, which obtained AR ChIP-seq data from 100 fresh frozen, high tumor cell percentage samples with known biochemical recurrence outcome, which have been validated in TCGA cohort. Stelloo, Nevedomskaya, Kim et al. Nature Communications 2018.
Data exclusions	Excluded samples and data have been reported in Stelloo, Nevedomskaya, Kim et al. Nature Communications 2018 for low sequencing quality, not pre-determined exclusion criterium.
Replication	Cas9 enhancer cut experiments for CITED2 were replicated by biological triplicates and by another replicate at another time-point. Not all replicates gave conclusive data. Therefore, we used an orthogonal method (Suntag-KRAB), which validated these findings independently through repression (CRISPRi) instead of DSB introduction by simultaneously using two sgRNAs.
Randomization	Patients from ranked ARBS were allocated in case and control groups based on biochemical recurrence outcome. Stelloo, Nevedomskaya, Kim et al. Nature Communications 2018. Outcome site patient groups were determined by the ratio of good:poor outcome sites as reported previously in Stelloo et al. EMBO Mol Med 2015.
Blinding	Blinding was not possible for case and control groups, as these were identified by biochemical recurrence outcome in the study design. Tumor samples, pathology and histology samples have been processed blindly during collection, during which supervisors were blinded (ref Stelloo, Nevedomskaya, Kim et al. Nature Communications 2018.). Chromogranin and synaptophysin immunoexpression were assessed using a routine optical microscope by an experienced pathologist blinded to molecular data. During analyses, investigators were not blinded to group allocation.

Reporting for specific materials, systems and methods

We require information from authors about some types of materials, experimental systems and methods used in many studies. Here, indicate whether each material, system or method listed is relevant to your study. If you are not sure if a list item applies to your research, read the appropriate section before selecting a response.

Materials & experimental systems

n/a	Involved in the study	
[ ]	[x]	Antibodies
[ ]	[x]	Eukaryotic cell lines
[x]	[ ]	Palaeontology and archaeology
[x]	[ ]	Animals and other organisms
[x]	[ ]	Human research participants
[x]	[ ]	Clinical data
[x]	[ ]	Dual use research of concern

Methods

n/a	Involved in the study	
[x]	[ ]	ChIP-seq
[x]	[ ]	Flow cytometry
[x]	[ ]	MRI-based neuroimaging

Antibodies

Antibodies used	Western blot:<br>Cas9 mouse (Cell Signaling #14697, clone 7A9-3A3, diluted 1:1000),<br>actin mouse (Sigma Aldrich A2228, clone C4, diluted 1:1000)<br>Flow Cytometry: Invitrogen MA5-38715, EPCAM-APC Clone 323/A3, diluted 1:200<br>ChIP: HOXB13 rabbit (Santa Cruz Biotechnology, sc-66923. clone H-80, 5ug per 50ul Protein A Dynabeads Invitrogen)
Validation	Cas9: 293T cells, mock transfected (-) or transfected with a construct expressing Cas9 (+). Horvath, P. and Barrangou, R. (2010) Science 327, 167-70.<br>Actin: HeLa whole cell lysate, muscle homogenates (MQ100 quality level) Otey 1987 and Wit et al. 2015 Nucleic Acids Research 2015; PMID 25505145<br>Both antibodies are used in many publications.<br>EPCAM-APC datasheet: https://www.thermofisher.com/order/genome-database/dataSheetPdf?productType=antibody&productsubtype=antibody_primary&productId=MA5-38715&version=233

HOXB13 used in Takeda et al. 2018 Cell: A Somatically Acquired Enhancer of the Androgen Receptor Is a Noncoding Driver in Advanced Prostate Cancer, doi:10.1016/j.cell.2018.05.037.

Eukaryotic cell lines

Policy information about cell lines

Cell line source(s)	LNCaP cells (ATCC, CRL-1740)<br>HEK293T cells (ATCC, CRL-3216)<br>MDA-MB453 (ATCC HTB-131)
Authentication	STR profiling
Mycoplasma contamination	All cells were regularly tested for mycoplasma contamination using PCR with appropriate controls, which showed cells were negative for mycoplasma.
Commonly misidentified lines<br>(See ICLAC register)	No commonly misidentified cell lines were used in the study.
