# Supplementary figures and images for: Extensive androgen receptor enhancer heterogeneity in primary prostate cancers underlies transcriptional diversity and metastatic potential
Source: Nat Commun. 2022 Nov 30;13:7367. doi: 10.1038/s41467-022-35135-2 (PMC9712620; doi:10.1038/s41467-022-35135-2)

Uncropped blots and gels

Figure 4E

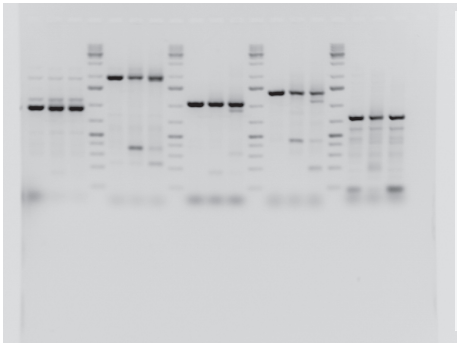

SFig. 7A

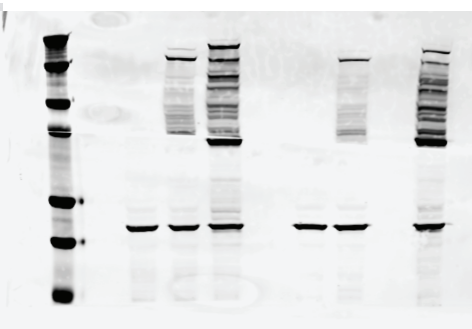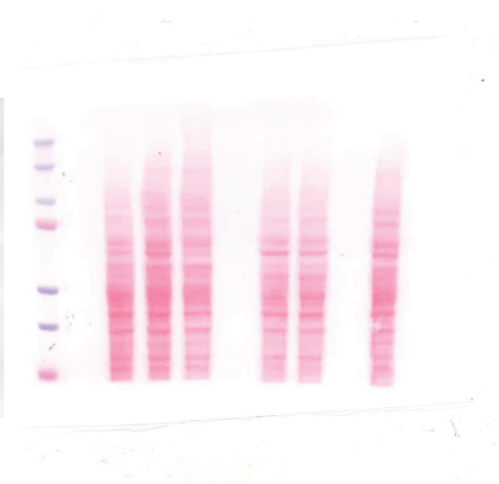

SFig 7D

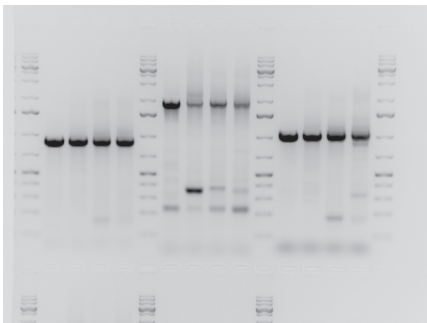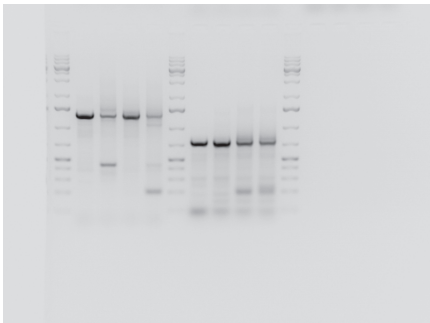

SFig 7E

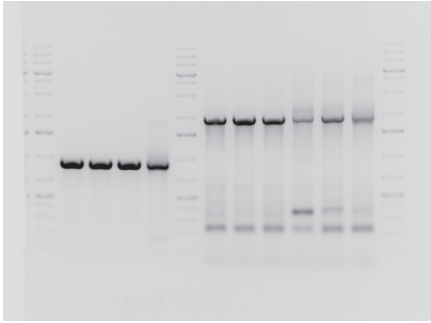

SFig 7H

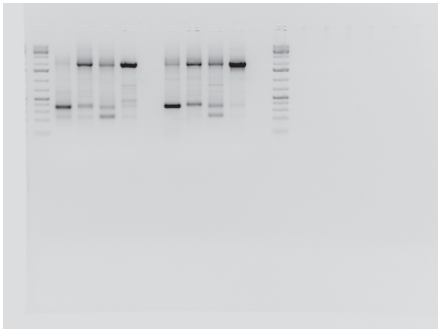

Supplement: Supplementary file 6 — Source Data [file 41467_2022_35135_MOESM6_ESM.zip › Uncropped blots and gels.pdf]
